# A New Perspective on Overfeeding in the Intensive Care Unit (ICU): Challenges, Dangers and Prevention Methods

**DOI:** 10.3390/life15050828

**Published:** 2025-05-21

**Authors:** Vlad-Dimitrie Cehan, Alina-Roxana Cehan, Mihai Claudiu Pui, Alexandra Lazar

**Affiliations:** 1 Anesthesiology and Critical Care Clinic, Emergency Clinical County Hospital of Targu Mures, 540139 Targu Mures, Romania; 2 Doctoral School of Medicine and Pharmacy, “George Emil Palade” University of Medicine, Pharmacy, Science and Technology of Tirgu Mures, 540142 Targu Mures, Romania; 3 Plastic and Reconstructive Surgery, Emergency Clinical County Hospital of Targu Mures, 540139 Targu Mures, Romania; 4 Anesthesiology and Intensive Care Department, “George Emil Palade” University of Medicine, Pharmacy, Science and Technology of Tirgu Mures, 540142 Targu Mures, Romania

**Keywords:** overfeeding, intensive care unit, ICU, nutrition support, metabolic complications, energy expenditure, indirect calorimetry

## Abstract

Overfeeding, currently defined as providing excessive energy and nutrients beyond metabolic requirements, is a common yet often overlooked issue in the intensive care unit (ICU) setting. Understanding the factors contributing to overfeeding and implementing strategies to prevent it is essential for optimizing patient care in the ICU. Several factors contribute to overfeeding in the ICU, including inaccurate estimation of energy requirements, formulaic feeding protocols, and failure to adjust nutritional support based on individual patient needs. Prolonged overfeeding can lead to insulin resistance and hepatic dysfunction, exacerbating glycemic control, increasing the risk of infectious complications, and worsening clinical outcomes. Clinically, overfeeding has been linked to delayed weaning from mechanical ventilation, prolonged ICU stay, and increased mortality rates. Regular review and adjustment of feeding protocols, incorporating advances in enteral and parenteral nutrition strategies, are essential for improving patient outcomes. Clinicians must be proficient in interpreting metabolic data, understanding the principles of energy balance, and implementing appropriate feeding algorithms. Interdisciplinary collaboration among critical care teams, including dieticians, physicians, and nurses, is crucial for ensuring consistent and effective nutritional management. Overfeeding remains a significant concern in the ICU after discharge as well, implying further complications for patient safety and integrity. By understanding the causes, consequences, and strategies for the prevention of overfeeding, healthcare providers can optimize nutrition therapy and mitigate the risk of metabolic complications. Through ongoing education, interdisciplinary collaboration, and evidence-based practice, the ICU community can strive to deliver personalized and precise nutritional support to critically ill patients.

## 1. Introduction

Existing healthcare methods for meeting patients’ needs to reintegrate into a functional and healthy life still face numerous challenges. Following discharge from the ICU (Intensive Care Unit), many patients do not resume their previous habits and instead transition to rehabilitation centers or assisted living facilities. This raises questions about the impact on their QoL (Quality of Life) post-discharge. [1] The changes in mental health, cognitive function, and physical well-being that persist after discharge from the intensive care unit are collectively referred to as Post-intensive Care Syndrome (PICS). This increases the overall recovery cost and necessitates ongoing care by healthcare providers. Since 2012, ICU societies and government institutions have been investing considerable resources in research on the QoL after ICU discharge. [2] Reducing post-discharge costs presents a significant challenge, as advanced procedures and therapies may be required. Therefore, clinicians must implement cost-effective practices and optimize therapeutic procedures to continuously improve post-ICU QoL. According to van Zanten et al. [1], nutritional support is critical in caring for critically ill patients, as it aims to meet energy requirements, preserve lean body mass, and enhance immune function.

Parenteral Nutrition (PN) represents an intravenous (IV) method of delivering essential macro- and micronutrients and fluids directly into the systemic circulation, therefore bypassing the enteral route. It is indicated when oral or enteral nutrition is insufficient, contraindicated, or physiologically nonviable due to gastrointestinal dysfunction or failure. Preserving the gut and gut immunity implied, considering PN side effects, resulted in clinicians preferring EN (Enteral Nutrition) for medical practice. Most recent ASPEN (American Society for Parenteral and Enteral Nutrition) guidelines underline the importance of energy overfeeding prevention when nutrition is administered via PN (Parenteral Nutrition). Based on several relevant clinical trials, current guidelines concluded that prevention of overfeeding energy, proper catheter management, and tight glycemic control may even lower the chances for bacteremia and hyperglycemic complications [3].

During the early days of PN, patients were frequently overfed; the term used is “hyperalimentation”. The complications found for patients receiving up to 5000 kcal/d were increased MV (minute volume), high pCO2 values, and more DOMV (days of mechanical ventilation) [4]. Recently, the term “hormesis” has gained traction, supporting the procedure in which a low quantity of a particular substance can have positive effects. On the other hand, high quantities can prove detrimental, leading to the concept of permissive underfeeding, which, as studies have demonstrated, improves outcomes and reduces the number of short- and long-term complications [5]. Permissive underfeeding is defined as a controlled nutritional strategy in critically ill patients that deliberately maintains caloric intake below full energy requirements (typically <70% of calculated energy needs) for a limited period, to mitigate metabolic stress without inducing significant protein catabolism or malnutrition [6].

Enteral Nutrition (EN) represents the delivery of nutrients directly into the gastrointestinal (GI) tract via oral supplements or tube feeding (e.g., nasogastric, gastrostomy, or jejunostomy tubes). It is recommended when oral intake is inadequate, but the GI tract remains functional. EN formulations contain balanced macronutrients (proteins, carbohydrates, fats), micronutrients (vitamins, minerals), and fluids, tailored to metabolic needs. Indications include dysphagia, critical illness, malabsorption, or neurological impairment. Unfortunately, EN has been proven unable to supply the necessary protein targets, especially in critical moments during critical illness, a fact derived from the complications of EN, as this meta-analysis demonstrated [7]. Puthucheary et al. studied the impact of EN on protein intake and nutritional supplements, observing that providing higher amounts than 1.5 g/kg/day was not possible while implementing EN alone, underlining the need for further studies [8]. There is a need for further insights into proving if slowly progressive enteral nutrition brings benefit compared to high-dose early EN or even low-dose early PN [9]. Clinicians must work with nutritionists to tailor early enteral nutrition for the patient, as well as pathology and preexisting conditions. ICU patients, being more exposed to conditions like sepsis or MSOF (Multiple Systemic Organ Failure), are associated with extended periods of mechanical ventilation, hospital stay, and increased costs of healthcare.

Acute liver failure (ALF) is a life-threatening condition defined by severe hepatic dysfunction (transaminase elevation, INR > 1.5, encephalopathy, etc.) without pre-existing chronic disease. Nutritional factors play a pivotal role in both triggering and managing ALF, as malnutrition exacerbates hepatic encephalopathy and coagulopathy while specific dietary toxins (e.g., herbal supplements, aflatoxin-contaminated foods) directly cause liver injury. Although paracetamol overdose and viral hepatitis are leading etiologies, 20–30% of cases remain idiopathic, highlighting the need for early nutritional assessment and intervention [10].

The scope of the article is to present the causes, changes in metabolism and also the consequences of overfeeding based on the main current articles and guidelines found in the literature, underlining the major changes in metabolism, taking into consideration the main phases of critical illness, while emphasizing the impact on nutritional support after admittance. Knowing the dangers of overfeeding and the means for prevention, clinicians are able to personalize nutritional interventions to particular pathologies to improve outcomes. Understanding the factors contributing to overfeeding and implementing strategies to prevent it is essential for optimizing patient care in the ICU [11].

## 2. Protein Pathway and Metabolic Changes

The catabolic state characterizes critical illness associated with an accelerated muscle mass loss caused by mass-scale proteolysis—the importance of activating the ubiquitin–proteasome pathway at the very core. Therefore, the main objective of achieving nutritional balance in the intensive care unit involves protein intake. Critical patients suffer metabolic alterations shifting toward catabolic processes. At the cornerstone of these processes stands the hypothalamus and the pituitary glands, stimulating the secretion of hormones like GH (growth hormone), TSH (thyroid-stimulating hormone), and cortisol. Reduced levels of ghrelin, linked with an increase in YY peptide together with cholecystokinin, promote anorexia in critical ICU patients [5].

Anabolic resistance refers to the inability of typical anabolic cues to stimulate messenger RNA (ribonucleic acid) translation of cellular protein, a relatively common phenomenon in critical illness. This resistance is believed to stem from compromised responses to amino acids, diminished insulin-mediated reduction in catabolism, sequestration of amino acids in the splanchnic segment, and decreased availability of muscle amino acids. mTOR proteins are crucial regulators of cell growth and metabolism, involving two distinct complexes, mTORC1 and mTORC2, which respond to various environmental signals such as nutrients, growth factors, and oxidative stress by upregulating glycolysis, lipogenesis, and amino acid uptake, all while downregulating catabolic pathways. mTORC1 is responsible for driving cellular anabolism, triggering autophagy, while MTORC2 modulates cytoskeletal dynamics [12]. Dysregulation of mTOR signalling contributes significantly to pathologies such as cancer (via unchecked proliferation) as well as metabolic disturbances (e.g., insulin resistance derived from aberrant mTORC2-AKT signalling) [13]. Rapamycin (mTORC Inhibitor) downscales excessive inflammatory process early in sepsis while escalating catabolism [14]. Conversely, Leucine (a branched-chain amino acid) is a critical activator of mTOR, integrating nutritional and growth factor signals (e.g., insulin-like growth factor-1, IGF-1) to promote anabolism [15,16]. However, in critical illness, protein metabolism is disrupted at multiple levels, including impaired autophagic vacuole maturation (62% reduction, *p* = 0.05) and a 97-fold increase (*p* < 0.03) in proteolytic activity, as demonstrated by Vanhorebeek et al. [17]. The stress response triggers excessive counterregulatory hormone secretion (corticosteroids, catecholamines, glucagon, and growth hormone), while proinflammatory cytokines (particularly TNF-α and IL-1) contribute to transient insulin resistance. This metabolic dysregulation impairs hepatic gluconeogenesis and peripheral glucose utilization in skeletal muscle. Multiple iatrogenic factors—including vasopressor therapy, glucocorticoid administration, and nutritional support—further modulate glucose homeostasis in this vulnerable population [18,19]. Metabolic stress is a concerning process that alters metabolism in critical patients suffering from burns, traumatic injuries, or sepsis. Multiple pathways are activated to ensure the nitrogen balance, preservation of muscle mass, and protein balance in the bloodstream [20]. Patients receiving protein supplementation above their estimated nutritional goal are vulnerable to hepatic steatosis and azotemia, predisposing them to immunosuppression. Extended bed rest triggers proteolysis due to a hypercatabolic state, and once the nitrogen imbalance is established, anabolic resistance accelerates continuous muscle loss [6,16]. The process is further amplified for patients suffering from MODS who can lose up to 1 kg of body muscle mass/day, considering only the first ten days from admission [8]. Severe inflammation encountered in acute-phase critical illness, leading to a limitation of metabolic tolerance, may be detrimental as it may increase the risk of overfeeding. In later phases, anabolism, characterizing the evolution toward chronic illness and recovery, increases the risk of underfeeding [21].

Critical illness induces a triphasic metabolic response characterized by distinct endocrine and inflammatory alterations. The acute phase (0–72 h) features hypothalamic–pituitary–adrenal axis activation with glucocorticoid-induced proteolysis and hepatic gluconeogenesis, catecholamine-mediated hypermetabolism, and cytokine-driven insulin resistance, therefore necessitating hypocaloric nutrition to prevent metabolic overload. The transitional phase (3–10 days) shows growth hormone-mediated partial metabolic recovery with persistent oxidative stress. During the transitional phase, partial insulin sensitivity restoration coincides with persistent catabolism and mitochondrial dysfunction, exhibiting mTOR pathway reactivation and insulin sensitivity normalization in favor of lean mass accretion; this phase requires nutritional advancements. The recovery phase (>10 days) holds anabolic predominance while restoring protein synthesis pathways, permitting an aggressive approach of nutritional repletion. Clinical nutritional management must account for this dynamic metabolic progression while monitoring for complications including hyperglycemia, refeeding syndrome, and hypertriglyceridemia [21].

## 3. Consequences of Overfeeding

**Figure 1:**
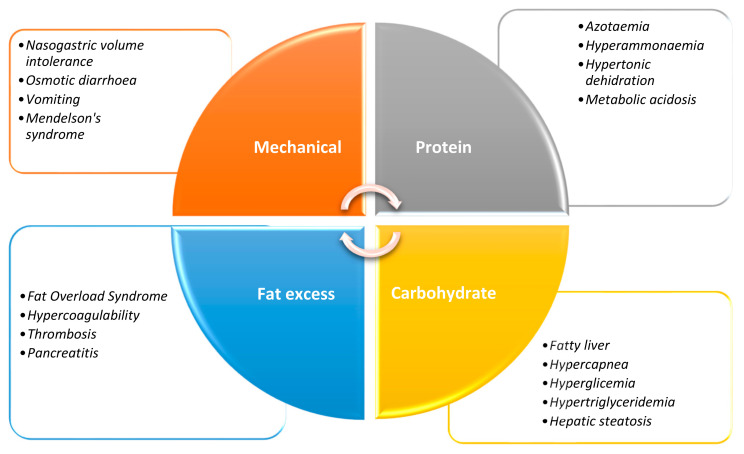
Mechanical and metabolic complications of overfeeding.

Overfeeding in the ICU is associated with a range of adverse consequences (Figure 1), both metabolic and clinical. Critical patients may frequently face gastrointestinal dysfunction at some point during their admission, such as gastroparesis and GI (gastrointestinal) dysmotility; all these complications are associated with increased DOMV (days of mechanical ventilation) and LOS (length of stay) [9].

Trials such as EAT-ICU or TARGET give insight into the early intake of a high-calorie diet, leading to hyperglycemic states in patients receiving fast-acting insulin in high doses [22,23]. The Early Goal-Directed Nutrition vs. Standard Care in Adult ICU Patients trial, a single-centered, randomized study, having included 203 adult ICU patients with ≥3 days of mechanical ventilation, revealed a group that received early aggressive nutrition, achieving significantly higher energy (92% vs. 73%) and protein (80% vs. 60%) intake vs. standard care (*p* < 0.001), but considerable higher rates of hyperglycemia that required insulin *p* = 0.03.

Studying glycemic control while using a clinically confirmed model of glucose-insulin, one single-centered study analyzing the 2011–2018 year period, with 1147 glycemic control procedures in the course of 8 h or more found a decrease in insulin sensitivity by 50–55% for the first four years period, low values being constantly recorded for 2015–2018 [24]. Analyzing glycemic balance, one Taiwanese cohort study on 29,084 patients revealed that hypoglycemia (<70 mg/dL) significantly increased mortality risk (48.9% vs. 15.9%, *p* < 0.001), with graded associations for severity (moderate aHR 1.48; severe aHR 1.85) and recurrence (single aHR 1.50; multiple aHR 1.61). Non-diabetics showed greater vulnerability than diabetics (*p* < 0.001), particularly for spontaneous episodes (53.9% vs. 42.4% insulin-related mortality). These findings demonstrate hypoglycemia’s independent prognostic value, with mortality risk modulated by glycemic status, etiology, and exposure characteristics [25]. Sheila e Harvey et al., studying 2400 patients receiving parenteral vs. enteral feeding admitted in 33 ICU units, found significantly lower rates of hypoglycemia (3.7% vs. 5.2%, *p* < 0.006) and vomiting (8.4% vs. 16.2%, *p* < 0.001), failing to demonstrate a difference in the 90-day mortality (37.3% vs. 39.1%, *p* = 0.72) [26].

Studying enteral nutrition tolerance in septic shock, in a single-centered retrospective cohort study, Qi F et al. found that the dose of norepinephrine (>0.3 μg/kg/min is related to EN intolerance (*p* < 0.001) due to splanchnic vasoconstriction with a threshold of >0.5 μg/kg/min for EN failure and higher 28-day mortality (42% vs. 24%; *p* = 0.01) [27]. Merriweather et al. found in a cohort of 193 patients an association between loss of appetite, based on a visual analogue scale, and length of stay with an inflammatory state documented by elevated serum CRP (C-reactive protein) levels [28]. Aiko Tanaka et al. studied the complications of overfeeding by administration of more than 30 kcal/kg/day; the results revealed frequent diarrhea (*p* = 0.02) and higher quantities of fast-acting insulin administered, finding also an increase in minute ventilation (Spearman Rho 0.27, *p* < 0.007). Another post hoc analysis from a small sample RCT comparing high protein intake in the first week with standard care demonstrated an increasing mortality rate when aggressive protein intake is implemented [29]. Studies have shown that overfeeding and underfeeding beyond that threshold leads to a rise in the mortality rate, which can be calculated as a U-shaped relation [30,31].

## 4. Strategies for Prevention of Overfeeding in Critical Care

### 4.1. Individualized Nutrition Therapy

Preventing overfeeding in the ICU requires a multifaceted approach, addressing both systemic and patient-specific factors (Table 1). Individualized nutrition therapy is paramount based on frequent monitoring of energy expenditure and metabolic parameters. Regular review and adjustment of feeding protocols, incorporating advances in enteral and parenteral nutrition strategies, are essential for optimizing patient outcomes. Failing to apply the guidelines leads to interruptions and disruptions in nutritional supplementation, a process amplified by delayed gastric emptying. Non-adherence to the implementation of prescribed doses of nutrition is an important factor that leads to a lack of improvement in the outcome of critical patients. According to the 2019 ESPEN (European Society for Parenteral and Enteral Nutrition) guidelines, enteral nutrition can be initiated right after the primary phase of successful hemodynamic stabilization [32]. A study on a reduced sample of traumatic brain injury patients supports this conclusion. Reduced rates of infection and a downscaled postinjury inflammatory response were obtained while reaching the estimated energy target starting at the moment of admittance, compared to a gradual increase during the first week in the ICU [33].

### 4.2. Monitoring and Assessment Tools

Utilizing indirect calorimetry to assess energy requirements in critically ill patients can help tailor nutritional support and minimize the risk of overfeeding. Following the latest guidelines is imperative, and monitoring overfeeding can be implemented by analyzing the expired gas. The respiratory quotient reaching a value R/Q > 1 indicates a state of overfeeding, while a respiratory quotient value of 0.8 to 1.0 is a strong indicator of increased CO_2_ due to protein metabolism. Using indirect calorimetry in certain target groups of patients is essential [35]. The tight calorie control study (TICACOS) sampled mechanically ventilated patients receiving indirect calorimetry-guided nutrition based on the REE value. Patients receiving nutrition based on the CI measurements received consistently more nutrition compared to patients receiving nutrition based on energy target calculations. The results revealed a downward trend regarding hospital mortality (32.3% vs. 47.7%, *p* = 0.058), while DOMV and ICU stays increased (*p* = 0.03 and *p* = 0.04) [36]. Consequently, increasing needs for metabolic substrate and higher values of mREE (measured resting energy expenditure) amplify the role of energy and nutrient therapy. Failure to adapt to these requirements leads to muscle-mass loss, reduced functional recovery, and altered physical abilities [37]. Inadequate monitoring of energy expenditure, reliance on predictive equations, and challenges in assessing nutritional status bring further complexity. Critical patients have an accelerated process of endogenous energy production, accomplishing their caloric needs, and observing that therapeutic nutritional measures cannot downscale this defensive mechanism of the critically ill [38].

### 4.3. Phased Nutritional Interventions

Nutritional support during critical illness evolves across different phases to address changing metabolic demands and support recovery. According to the ASPEN guidelines, critically ill patients generally benefit from a protein intake of 1.2–2.0 g/kg/day. During the acute phase, energy and protein delivery should be gradually increased to meet 80–100% of energy expenditure and 1.3 g/kg/day of protein by Day 3 to minimize the risk of overfeeding complications [3,39]. Allingstrup et al. concluded that increasing the quantity of proteins delivered in a dose-dependent ratio improves survival rates [40]. Several changes to nutritional supportive methods mark the post-acute phase of critical illness. Therefore, the recommended calorie intake could rise to 125–150% of the estimated values. The target for protein consumption may be safely adjusted to 1.5–2.5 g per kilogram of body weight per day [1].

Special considerations are required for elderly patients over 65 years of age, as key differences exist between ASPEN and ESPEN guidelines. ASPEN recommends an energy intake of 22–25 kcal/kg/day, while ESPEN suggests a more conservative target of 18–22 kcal/kg/day. Furthermore, assessment methods also differ, with ASPEN favoring tools like handgrip strength and the Short Physical Performance Battery (SPPB), whereas ESPEN emphasizes biochemical markers such as C-reactive protein (CRP) and albumin [3,41].

For patients with thermal injuries, individualized therapy is essential due to the unique metabolic demands. ASPEN supports restrictive energy delivery of ≤20 kcal/kg/day during the initial 72 h resuscitation phase of major burns, prioritizing protein retention with 1.5–2.0 g/kg/day through enteral nutrition. In the subsequent transitional phase, energy provision should progressively escalate to 25–30 kcal/kg/day, with protein targets increasing to 2.0–2.5 g/kg/day to mitigate nitrogen wasting. ESPEN protocols similarly emphasize energy delivery of 20–25 kcal/kg/day and protein intake adjusted based on wound severity, ranging from 1.5 to 2.5 g/kg/day. Continuous metabolic monitoring through indirect calorimetry is critical, particularly for patients with burns covering more than 30% of the total body surface area [3].

In the chronic phase of critical illness, defined as more than seven days post-admission, patients typically regain their ability to process nutrients effectively. This phase is a pivotal period where nutrition significantly contributes to recovery (Table 2). Adjustments to energy and protein intake are made to support tissue repair, immune function, and overall rehabilitation, aligning with the patient’s metabolic capacity and clinical progress [36].

Vankrunkelsven et al. [44] set out to inquire about current practices regarding minerals and micronutrients. Medical professionals who responded did not measure blood samples for nutrients daily, and no protocol regarding monitoring had been implemented. Critical Care Patients are exposed to a series of factors that lead to anemia; speculations revolve around the deficiency of the following micronutrients: folate, vitamin B12, and iron; therefore, in selected cases, the three are currently recommended to be supplemented. Vitamin B1 has been used to prevent cardiac failure and Wernicke encephalopathy [45]. Vitamin C has been supplemented along with thiamine as metabolic adjuvants for septic shock patients [46]. Doig GS et al. studied temporary macronutrient restriction, finding improved survival while correcting micronutrient deficits and increasing macronutrient dosage [47].

### 4.4. Complications and Mitigations

Quantitative reduction in nutritional support should not expose the patient to complications such as Refeeding Syndrome, which occurs after a prolonged lack of phosphate, potassium, vitamin B1, electrolytes, and other micronutrients. Risks include cardiac failure, arrhythmias, respiratory failure, or lactic acidosis [17,48]. Managing refeeding syndrome patients may be challenging, and one study enrolling 339 critical patients suggests that caloric restriction improves 60-day survival rates (*p* = 0.002) and overall survival time (*p* = 0.002) [47].

The application of BCAAs for managing liver failure and hepatic encephalopathy is grounded in their ability to improve ammonia detoxification in muscle tissue and support liver regeneration [49]. Holecek et al. observed that supplementing nutrition with BCAAs (branched-chain amino acids) could increase the production of ammonia from glutamine metabolism in the gut and kidneys, potentially exacerbating hepatic encephalopathy [50]. A prior study found that an arginine-enriched diet for perioperative patients led to fewer infections and a shorter hospital stay, but it did not significantly impact mortality rates [51]. Low concentrations of glutamine have been linked to muscle wasting and poor outcomes in critical care patients, being considered essential for hepatocytes, enterocytes, and other immune cells to function. Nutritional management for these patients resembles that for individuals with sarcopenia or secondary cachexia, with a recommended protein intake of 1.5–2.0 g/kg/day potentially being suitable [52]. Trials like Reducing Deaths Due to Oxidative Stress (REDOXS) have supplemented 0.6 to 0.8 g/kg/day of glutamine and found an increase of 6.5% in the risk of death after 28 days and after 6 months for organ failure patients receiving enteral glutamine treatment and PN nutrition in high doses [53]. Stehle et al. [54] conducted a meta-analysis of 15 RCTs on 842 hemodynamically stable patients, excluding those with liver or renal failure. Their findings indicated that parenteral glutamine dipeptide supplementation notably reduced infectious complications, ICU and hospital length of stay, mechanical ventilation duration, and hospital mortality by 45%, though it did not affect ICU mortality [54].

### 4.5. Emerging Evidence

NUTRIREA-3 (2023), the latest RCT studying mechanically ventilated patients receiving vasopressors, found that lower targets for energy and protein dosage administered through EN and/or PN (6 kcal/kg/day and 0.2–0.4 g/kg/day) compared to the common practices (25 kcal/kg/day and 1.0–1.3 g/kg/day) can be associated with decreased frequency of complications such as diarrhea, vomiting, and event mesenteric ischemia caused by EN and also a significant reduction in LOS [55]. Data show that, on average, only 60% of critical patients meet the requirements for protein intake [16]. Achieving a nutritional value of 80% of protein delivery shows a promising result with a decrease in days of DOMV, LOS, and higher survival rates in the ICU. In the Early Parenteral Nutrition completing Enteral nutrition in Adult Critically ill Patients (EPaNIC) trial, patients receiving late parenteral nutrition >day 7 and IV glucose increased the chance of live discharge percentage by 6.3% (*p* < 0.05), as well as the opportunity for live discharge as well as early discharge from the ICU unit [56].

A more recent study implemented on pig subjects studying the effects of continuous enteral nutrition (a period > 24 h) observed that proteins administered via enteral feeding remain in the splanchnic blood vessels, the gut moderating the transport of proteins out of its circulation [57]. Gazzaneo MC studied intermittent feeding on neonatal pigs, revealing that protein synthesis doubled in quantity compared to intermittent and continuous feeding. Studying animal models, the authors indicate that intermittent nutrition administration, in contrast to continuous administration, enhances protein synthesis and various other outcomes. These include the preservation of autophagy and the maintenance of the neurohormonal response to luminal nutrients [58].

According to a retrospective cohort study published by Nicolo et al. based on critically ill patients admitted for more than 4 days (2828 patients) with a subsample of >12 days LOS group (1584 patients), achieving more than 80% of prescribed energy reduced mortality in the >4-day admission population (*p* < 0.01), also establishing that protein intake >1.2 g/kg/day correlates with survival (*p* < 0.05) [59]. Piton G et al., in their NUTRIREA-2 trial, found a higher concentration of citrulline after more than 72 h (mass proportional to the enterocyte number) for patients who had received early EN, concluding that the gut mucosa can significantly benefit from an early start to EN [60]. While studying ways of delivering nutrition, Evans et al. conducted a study randomly assigning 60 critically ill adults to continuous or intermittent bolus enteral nutrition. Their findings indicated no significant variances in glycemic variability, insulin usage, tube feeding volume, or caloric intake between the two experimental groups [61].

**Figure 2:**
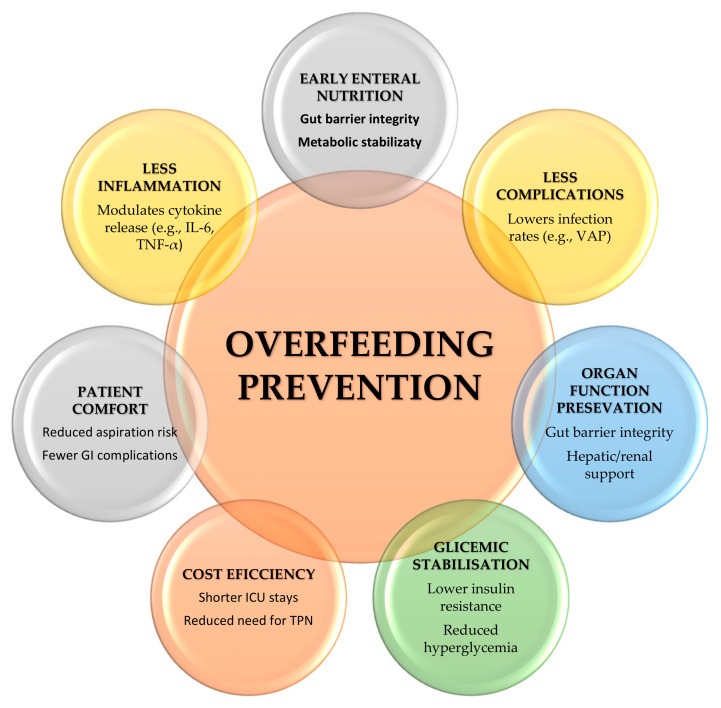
Challenges in overfeeding prevention.

Clinicians must be proficient in interpreting metabolic data, understanding the principles of energy balance, and implementing appropriate feeding algorithms (Figure 2). Interdisciplinary collaboration among critical care teams, including dietitians, physicians, and nurses, ensures consistent and effective nutritional management. ESPEN successfully identified scores that have the potential to screen and assess high-risk patients, underlining the importance of evaluating the patient’s nutritional balance and adapting the nutritional support to specific pathologies [3].

## 5. Nutritional Data After ICU Discharge

**Figure 3:**
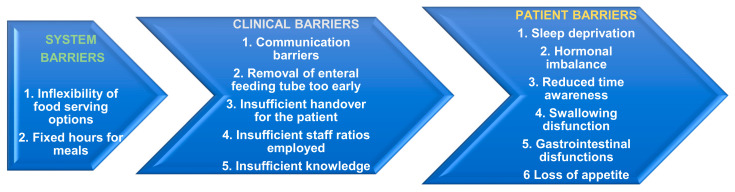
Barriers to assuring adequate nutritional support in the post-ICU period.

At this point, there are no official guidelines regarding recommendations on after-discharge nutritional guidance. Implementing clinical nutritional management may improve muscle mass loss significantly [23]. The effects of the acute phase of critical illness that impact the patient’s QoL cannot be denied, generating considerable healthcare costs and questions about the importance of nutrition in this matter (Figure 3) [62].

The oral route is the most common during the chronic phase of inflammation. Frequent complications met post-ICU are loss of appetite, taste modifications, difficulty chewing or swallowing, and cognitive impairment or inability to employ proper food services. Several studies report reduced nutritional intake in mechanically ventilated patients post-ICU discharge [63,64]. A study published in 2020 reported median post-discharge compliance with nutritional assistance prescribed by dieticians of 46% (IQR, 26–100%) for protein and 71% (IQR, 38–100%) for energy intake. Assessing the oral pathway for nutrition, the prescribed doses for protein 27% (15–41%) fall considerably compared to energy intake 47% (29–66%) [65]. The period right after discharge presents multiple challenges, represented by miscommunication, care management interruptions, and treatment interruptions [66].

Van Zanten et al. studied clinical nutrition, concluding that supplementation of energy during the chronic phase compared with the predicted value can reach up to 150%, clinicians may consider delivering 35 kcal/kg/day; at the same time, protein intake can safely surpass 2 g/kg/day, with benefits outweighing the risks at this stage [1]. The metabolic needs of critical patients in the recovery phase/chronic illness are estimated to be around 1.7 times resting EE [33]. At the 3-month follow-up of a series of subjects included in an RCT, physical performance improved significantly, enhancing their recovery with just a 25% increment in overall nutritional intake [67]. One prospective longitudinal study evaluated 174 acute lung injury survivors from the EDEN trial, assessing long-term physical (6 min walk distance, strength) and cognitive outcomes at 6 and 12 months post-ICU. While patients showed suboptimal recovery in both domains (36 vs. 25%, *p* = 0.001) with modest improvement over time, initial trophic versus full enteral feeding during the first 6 days of mechanical ventilation had no significant impact on these outcomes [68].

## 6. Education and Training

Comprehensive education and training programs for healthcare providers are essential to raise awareness of the risks associated with overfeeding and promote evidence-based nutrition practices in the ICU. The difference between the nutritional support of critically ill patients and healthy individuals must be made, understanding that nutritional support cannot manage critical illness. Therefore, a step-by-step approach to incremental volumes of EN and PN must be implemented [38]. A cohort study published in 2017 on 316 critically ill patient with GI pathology, demonstrated that the group of patients with GI dysfunction had lowered mortality while nutritional support when managed by a Nutritional support team (NST) and the transition to EN was practiced early had lowered mortality (12% vs. 21%, *p* = 0.03), shorter ICU LOS (median 8 vs. 11 days, *p* = 0.04), and fewer infectious complications [69].

Assessing nutritional status using the Mini Nutritional Assessment Score, Moradi et al. found a statistically significant association (*p* < 0.001) between weight loss and physical and neurophysiological dysfunctions with old age. Therefore, the risk must be assessed at admission to better identify the patients at risk for malnutrition [70]. The outcomes for critical patients have been observed to be improving for patients presenting at admission with considerable muscle mass. Overconsumption leading to obesity is a significant cause of metabolic syndrome, defined by high blood pressure, heart or cerebral stroke, and diabetes mellitus. Chronically overeating patients pose a challenge for glycemic balance in the ICU, being at high risk of developing type 2 diabetes. Obesity has also been linked with decreased cognitive function and memory; therefore, introducing high unsaturated fats such as nuts, fish, olive oil, or avocados may alleviate cognitive decline [71].

Multiple dysfunctions may have to be managed in the post-ICU phase so that enteral nutrition can be sufficient, according to the “SPICES concept”(Swallowing Dysfunction, Pancreatic/Intestinal Insufficiency, Inflammation/Metabolic stress, Coagulopathy/Thrombosis, Electrolyte imbalances, Sarcopenia/Immobility): disorders regarding swallowing have to be addressed, continuously screening the patients for PICS criteria, managing cases as a multidisciplinary team, collaborating with nutritionists and dieticians aiming toward an individualized approach to nutritional supplementation [63] (Table 3). Several assessment tools exist to evaluate swallowing function, typically involving the ingestion of controlled volumes with varying viscosities. While originally designed for non-ICU populations, certain validated methods—such as the Volume-Viscosity Swallow Test (V-VST)—have demonstrated safety and efficacy in acute care settings, including critically ill patients. These tools help clinicians identify aspiration risk and guide appropriate dietary modifications or alternative feeding strategies [72].

## 7. Conclusions

The future of critical care nutrition lies in precision medicine, where phase-specific protocols and real-time metabolic monitoring enable clinicians to tailor feeding strategies to each patient’s evolving needs. Advances in non-invasive monitoring now allow dynamic adjustments, minimizing overfeeding risks while optimizing recovery. Beyond the ICU, standardized rehabilitation programs are emerging to address Post-Intensive Care Syndrome, bridging gaps in long-term nutritional support. Research is breaking new ground in immunonutrition and gut microbiome modulation, challenging outdated paradigms and refining evidence-based guidelines. Together, these strides herald a transformative era in critical care, where survival is no longer the sole goal, but rather the restoration of function, dignity, and quality of life for ICU survivors.

## Figures and Tables

**Table 1:** Proposed Scores for Screening Critically Ill Patients for Malnutrition Based on Current Guidelines in Practice [3,34].

MST “Malnutrition Screening Tool”	MUST Malnutrition Universal Screening Tool	mNUTRIC Modified NUTrition Risk in the Critically Ill Score	Nutrition Risk Score 2002
Factors in: 1. Losing weight without trying, and how much 2. Decreased appetite 3. Appetite score	Parameters: 1. BMI 2. Weight lost without trying last 3–6 months 3. Acute illness	Calculating risk on a series of factors: 1. Age 2. Comorbidities 3. APACHE II score (Acute Physiology and Chronic Health Evaluation) 4. SOFA score (Sequential Organ Failure Assessment_ 5. Days of admittance in the facility before ICU 6. IL6 (interleukin-6) *	Risk calculated by factoring: 1. Weight loss 2. Low BMI 3. Low quantities of nutritional support 4. Disease severity
Requires patient cooperation, not fit for sedated patients	It does not take into consideration body composition	It does not factor in BMI as a primary parameter; it does not need patient cooperation	It does not take into consideration body composition, being based on BMI
It does not take into consideration age or practical aspects	Low specificity for critical patients	They are commonly used in practice for the target population at risk for malnutrition (intubated/sedated)	They are commonly used in practice for the target population at risk for malnutrition (intubated/sedated).
Rated as “good/strong”	Rated less reliable than mNUTRIC score	Supported by ASPEN/SSCM guidelines (2021)	Supported by ASPEN/SSCM guidelines (2021)

* The score may be calculated without introducing IL6 values.

**Table 2:** Current General Recommendations for Acute and Post-Acute Critical Illness Phases [3,42,43].

Nutrient	Acute Phase < 3d (2023)	Post-Acute Phase > 7d (2023)	Reasoning
Energy (Calories)	20–25 kcal/kg/day (or hypocaloric feeding in the first 7–10 days)	25–30 kcal/kg/day	Acute phase: Avoid overfeeding; hypocaloric feeding reduces complications like hyperglycemia and liver stress. Increase caloric intake to support recovery and rehabilitation.
Protein	1.2–2.0 g/kg/day (up to 2.5 g/kg/day for burns or severe trauma)	1.5–2.5 g/kg/day (up to 2.5–3.0 g/kg/day for severe muscle wasting)	Higher protein intake supports muscle repair, wound healing, and immune function.
Carbohydrates	50–60% of total calories		Acute: Provide a steady source of energy, avoid excessive intake; target blood glucose: 140–180 mg/dL.
Fats	25–40% of total calories		Emphasize omega-3 fatty acids (e.g., fish oil) for anti-inflammatory effects.
Fiber	Avoid or limit	25–30 g/day	Supports gut health and prevents constipation; avoid in cases of ileus or bowel obstruction.
Fluids	30–35 mL/kg/day		Adjust based on hydration status, renal function, and fluid losses.
Micronutrients	- Vitamin C: 500–1000 mg/day		Support immune function, wound healing, and metabolic processes.
	- Vitamin D: 1000–2000 IU/day		
	- Zinc: 15–30 mg/day		
	- Selenium: 50–100 mcg/day		
	- Magnesium: 200–400 mg/day		
Immunonutrition	- Glutamine: Avoid in severe sepsis/multiorgan failure	- Glutamine: 0.3–0.5 g/kg/day	Enhances gut integrity, wound healing, and inflammation modulation.
	- Arginine: Use cautiously		Immunonutrition is context-specific; avoid in certain conditions (e.g., severe sepsis).
	- Omega-3 Fatty Acids: 1–2 g/day		

**Table 3:** Future Lines of Research Regarding Overfeeding Prevention.

Research Focus	Key Questions	Expected Impact
Feeding Strategies	Low-rate vs. high-dose EN; EN vs. PN; patient-specific approaches	Optimize feeding protocols to improve outcomes and reduce complications.
Refeeding Syndrome and GI Tools	Define refeeding syndrome in the ICU; improve GI function assessment tools	Prevent complications and ensure safe nutritional support.
Protein Delivery	Protein targeting based on lean body mass	Refine strategies for complex, dynamic critical illness.
Biomarkers and Monitoring	Biomarkers for phase transition, overfeeding harm, and individualized nutrition	Enable precision nutrition and real-time adjustments in ICU care.
Sepsis and Repeated Episodes	Nutritional management during recurrent sepsis	Refine strategies for complex, dynamic critical illness.
Outcome Measures	Clinically relevant and patient-centered outcomes	Align research with patient needs and improve long-term recovery.

## Data Availability

The original contributions presented in the study are included in the article, further inquiries can be directed to the corresponding author.

## Abbreviations

The following abbreviations are used in this manuscript:

ALF	Acute Liver Failure
ASPEN	American Society for Parenteral and Enteral Nutrition;
BCAAs	Branched-Chain Amino Acids
BUNDOMV	Blood Urea NitrogenNumber of Days of Mechanical Ventilation
EN	Enteral Nutrition
ESPEN	European Society for Clinical Nutrition and Metabolism
mNutric	Validated tool to assess malnutrition risk in ICU patients (incorporates APACHE/SOFA scores).
REE	Resting energy expenditure
MODS	Multiple Organ Dysfunction Syndrome
mTOR	Mechanistic target of rapamycin
PICS	Post-Intensive Care Syndrome
PN	Parenteral Nutrition
RQ	Respiratory Quotient—CO_2_ produced/O_2_ consumed; RQ > 1 indicates overfeeding
SPICES	Framework for post-ICU nutrition: Swallowing, Pancreatic, Inflammation, Coagulopathy, Electrolytes, Sarcopenia.
SPPB	Short Physical Performance Battery
QoL	Quality of Life
